# The TG2/LRP1 Pathway for T Cell Activation by Post‐Translationally Modified Antigens

**DOI:** 10.1111/imr.70155

**Published:** 2026-08-04

**Authors:** Agnele Sylvia Sewa, Fu‐Chen Yang, Chaitan Khosla

**Affiliations:** ^1^ Department of Biochemistry Stanford University School of Medicine Stanford California USA; ^2^ Department of Chemistry Stanford University Stanford California USA; ^3^ Department of Chemical Engineering Stanford University Stanford California USA; ^4^ Sarafan ChEM‐H Stanford University Stanford California USA

**Keywords:** autoimmunity, celiac disease, dendritic cells, neo‐epitopes, post‐translational modification, TG2/LRP1 pathway

## Abstract

Post‐translational modifications (PTMs) can generate neo‐epitopes, modified peptides that evade immune tolerance and trigger immune responses. This review focuses on the TG2/LRP1 pathway as a new paradigm for coupling the formation of post‐translationally modified peptides with their effective presentation as T‐cell antigens by dendritic cells. Transglutaminase 2 (TG2) catalyzes the Gln → Glu conversion of specific residues in peptides derived from dietary gluten thereby enhancing their affinity for HLA‐DQ2, the principal genetic determinant of celiac disease. However, because such modified peptides are scarce in intestinal mucosa, an effective mechanism for lysosomal uptake by antigen‐presenting cells (APCs) is necessary. Protein–protein interaction between certain peptide‐bound TG2 complexes and the low‐density lipoprotein receptor‐related protein 1 (LRP1) results in efficient endocytosis of these antigenic peptides along with their concomitant release in the endo‐lysosomal compartment as deamidated products. An analogous PTM‐driven mechanism for antigen presentation may also operate in autoimmune conditions associated with peptidylarginine deiminase (PADI) activity, such as rheumatoid arthritis (RA). The exquisite cellular selectivity of the TG2/LRP1 pathway has implications not only for understanding its role in autoimmunity but also for its potential exploitation in vaccine design.

## 1 Introduction

In addition to epigenetic, transcriptional, and translational changes, post‐translational modifications (PTMs) that alter the covalent structures of selected amino acid residues of cellular and extracellular proteins represent a powerful class of biological regulatory mechanisms. While PTMs predominantly lead to changes that enhance the adaptive fitness of living systems, in some cases they induce, sustain, or propagate pathological states. In autoimmune diseases, for example, PTMs have been observed to enhance the accessibility of antigens to disease‐associated Human Leukocyte Antigens (HLAs).

The primary focus of this article is on a recently discovered mechanism for eliciting powerful T cell responses against antigens that have undergone a specific PTM. This mechanism involves coupling the enzymatic activity of extracellular transglutaminase 2 (TG2) with the endocytic activity of the low‐density lipoprotein receptor‐related protein 1 (LRP1) and is hereafter referred to as “the TG2/LRP1 pathway” [1, 2]. While the TG2/LRP1 pathway has direct relevance to celiac disease (CeD) autoimmunity, emerging data suggest that variations of this theme may contribute to the pathophysiology of other autoimmune disorders. If so, the underlying principles may not only be useful in designing therapies for CeD but could also be harnessed for enhancing the strength and/or longevity of immune responses to antigens of interest in vaccine development.

To set the stage for our discussion of the role of the TG2/LRP1 pathway in autoimmunity, we will first review how TG2 generates neo‐epitopes. For comparison, selected other mechanisms that generate PTMs of relevance to autoimmunity will be introduced. While the enzyme‐catalyzed formation of a neo‐epitope with enhanced T cell recognition characteristics is necessary to elicit an adaptive immune response, it is by no means sufficient. For a neo‐epitope to be efficiently loaded into a disease‐associated HLA in an antigen presenting cell, it must be transported into the subcellular compartment in which such an HLA‐peptide encounter can occur; this requires the ligand to cross one or more membrane barrier(s). We will therefore also compare mechanisms that facilitate such transport with an eye toward assessing their scope and limitations for certain types of antigens. In closing, our review of recent data and unanswered questions pertaining to the TG2/LRP1 pathway will be followed by a more speculative discussion of other related mechanisms that may be involved in coupling PTMs to autoimmune T cell activity.

## 2 Enzymes That Form Neo‐Epitopes and Mechanisms That Regulate Them

Immune tolerance is broken when the adaptive immune system mounts an inflammatory response to a “non‐self” peptide (neo‐peptide) derived from an endogenous or exogenous protein or peptide against which a central (thymic) or peripheral tolerogenic response was previously generated. Neo‐peptide biosynthesis sometimes occurs non‐enzymatically (e.g., glycation of hemoglobin via Schiff's base formation followed by Amadori rearrangement in response to elevated blood glucose levels, radical‐promoted cysteine oxidation in the context of inflammation, or time‐dependent deamidation of certain asparagine residues), although it is typically enzyme‐catalyzed. Because neo‐peptides must be selected by the adaptive immune system to become neo‐epitopes, enzymatic PTMs are the preferred route for neo‐epitope generation due to the inherent sequence specificity of most enzymes. Additionally, enzymatic PTMs can only occur in environments where the tightly regulated catalytic activity of the corresponding enzyme is expressed, thus limiting PTMs to specific organs and tissues and as responses to specific signals.

In this section we discuss the chemistry of certain PTM‐catalyzing enzymes known to play roles in neo‐epitope formation. We also highlight their allosteric regulatory features that play prominent roles in spatiotemporal control of neo‐peptide generation.

### 2.1 Gln Deamidation by Transglutaminase 2

**SCHEME 1:**
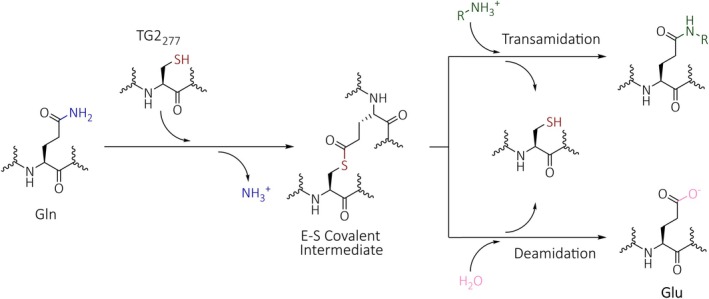
Deamidation and transamidation of transglutaminase 2.

The transglutaminase (TG) family of calcium‐dependent enzymes plays key roles in many biological functions including blood clotting, skin barrier formation, and crystallin crosslinking in the eye lens. The human genome encodes eight catalytically active TGs (Factor XIIIA, TG1, TG2, TG3, TG4, TG5, TG6, TG7) [3, 4]. TG2, which is possibly the most ubiquitous of all transglutaminases in the human body, catalyzes both transamidation and deamidation reactions (Scheme 1).

Since the discovery of TG2 as the primary autoantigen in CeD and demonstration that TG2‐catalyzed deamidation of gluten peptides enhances their ability to activate CeD‐specific inflammatory T cells in an HLA‐restricted manner, extensive literature supports a central role for TG2 in generating neo‐epitopes from dietary gluten to drive CeD autoimmunity. While TG2 is moderately specific for substrates harboring the Q‐X‐P sequence, a widespread motif in gluten proteins, unbiased approaches such as phage display have revealed that human TG2 can recognize a broader spectrum of Gln‐containing substrates [5, 6, 7, 8, 9]. When taken together with recent structural evidence that enzyme‐substrate complexes are predominantly stabilized by non‐covalent interactions involving the substrate peptide backbone, the need for additional mechanisms to explain how immunodominance in CeD patients tends to be restricted to relatively few TG2‐derived neo‐peptides becomes evident.

The most well‐understood of these immune selection mechanisms for converting neo‐peptides into neo‐epitopes is the HLA context. Specifically, onset of CeD obligately requires HLA‐DQ2 or HLA‐DQ8 to facilitate gluten antigen presentation to disease‐specific T‐helper (Th1) cells [10, 11]. Other selection mechanisms include proteolytic resistance of gluten peptides to gastrointestinal breakdown due to their high proline content, which allows certain partially digested peptides to persist in the small intestine for long enough to encounter TG2. As discussed below, the ligand specificity of the recently elucidated TG2/LRP1 pathway is also a powerful mechanism for neo‐epitope selection.

**FIGURE 1:**
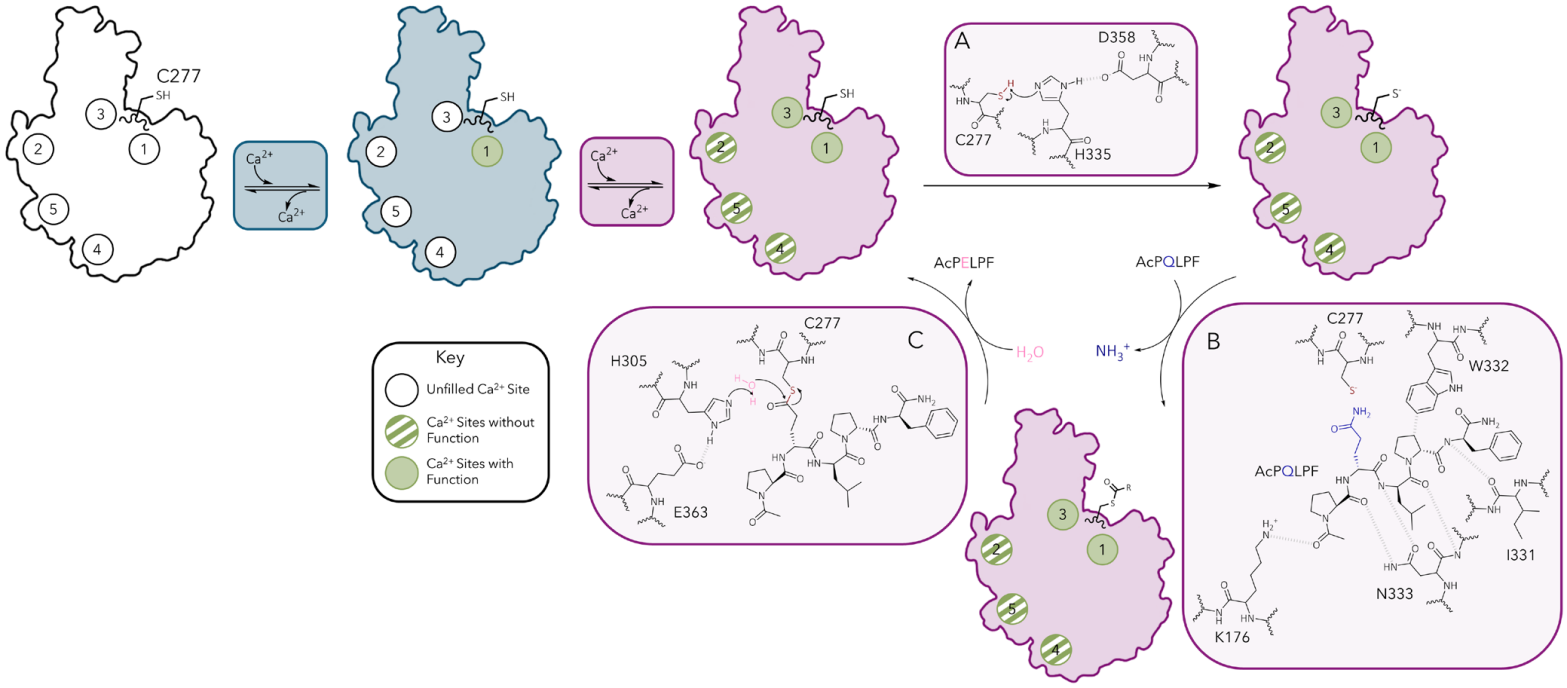
TG2 catalytic cycle. Calcium binding at the high affinity Ca^2+^ S1, shown in teal, prevents oxidation of TG2's vicinal disulfide bond. Subsequent calcium binding at low affinity calcium sites, specifically Ca^2+^ S3, allow TG2 to adopt the catalytically active states shown in purple. (A) Formation of the γ‐glutamyl thioester intermediate is initiated by deprotonation of the active site cysteine, C277, facilitated by H335 and D358. (B) The incoming substrate, AcPQLPF, is guided into the active site by I331, W332, N333, and K176, directing the glutamine sidechain in proximity to the activated cysteine. (C) H305 and E363 facilitates the deprotonation of the incoming nucleophile, H2O, allowing for subsequent hydrolysis of the γ‐glutamyl thioester intermediate and release of the deamidated product, AcPELPF.

Two Ca^2+^ ions play critical roles in regulating the enzymatic activity of TG2 (Figure 1). The high‐affinity site (Site1) is occupied at sub‐micromolar Ca^2+^ concentrations, although by itself it is incapable of activating the enzyme. Instead, its role appears to prevent premature oxidative deactivation as TG2 is exported into the extracellular environment by a non‐canonical (likely exosomal) mechanism. For TG2 to modify a peptide or protein substrate, the lower‐affinity Ca^2+^ site (Site3) must be occupied. Ca^2+^ binding is associated with a drastic enhancement of conformational flexibility of the two C‐terminal b‐barrel domains of the protein, allowing its catalytic core to form a thioester intermediate with a suitable substrate at its active site cysteine (C277). Deprotonation of C277 is facilitated by H335 and D358, while the substrate is anchored by hydrogen bonds between residues K176, I331, W332, and N333 and multiple backbone amide bonds in the substrate. Gln deamidation requires nucleophilic attack of this thioester intermediate by deprotonated water, presumably facilitated by H305 and E363 [12, 13].

Remarkably, although TG2 expression is ubiquitous in the mammalian small intestine and is abundantly distributed in the cytosol of epithelial cells as well as in the extracellular matrix of the intestine, a healthy intestine has virtually undetectable levels of enzymatic TG2 activity [2]. Therefore, spatial and temporal control of this tightly regulated PTM must play a critical role in both the initial loss of oral gluten tolerance in CeD as well as the recurrence of T cell inflammation in response to repeated gluten consumption by a patient.

### 2.2 Citrullination by Peptidylarginine Deiminases

**SCHEME 2:**
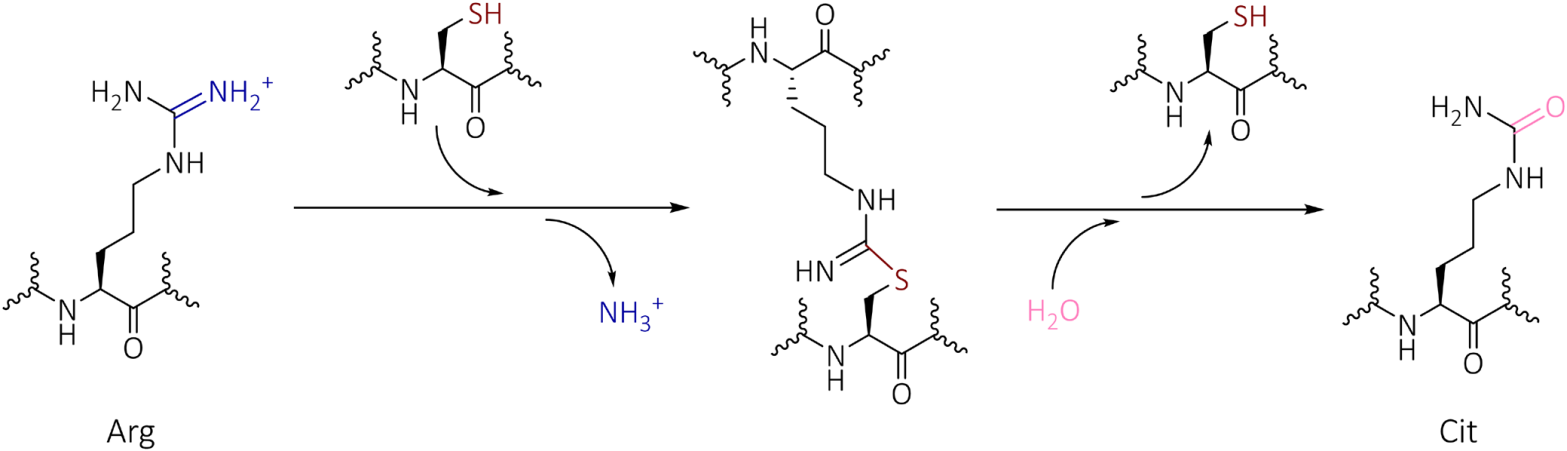
Citrullination by peptidylarginine deiminases.

Like transglutaminases, peptidylarginine deiminases (PADIs) are a family of Ca^2+^‐dependent enzymes with Cys active site residues [14, 15, 16, 17]. They catalyze the hydrolytic conversion of selected arginine residues into citrulline (Scheme 2). Five PADI homologs are encoded in the human genome and are differentially expressed in various parts of the body [18]. Notably, PADI enzymes also have tandem β‐barrel domains with uncanny structural (but not sequence) similarity to those of TG2, although these β‐barrel domains are fused to the N‐terminus of the catalytic domain.

Calcium binding not only affects PADI activity but also influences interactions with other protein partners. While PADI1, PADI2 and PADI6 do not show any obvious sequence specificity, the R‐G‐G motif is preferred by PADI4 [19]. A comprehensive analysis of 14,056 citrullination sites in 4008 proteins using PADI4 led to the identification of the 3Φ‐R‐G motif in its preferred substrates, where 3Φ represents three consecutive bulky hydrophobic residues [20].

Whereas the active site C645 of PADI4 is deprotonated by neighboring H471, PADI2 appears to utilize a substrate‐assisted catalytic mechanism to hydrolyze the guanidinium side chain of its Arg substrates [21]. Regardless of these mechanistic differences, all PADI enzymes convert a cationic residue into a neutral citrulline. In turn, citrullinated neo‐peptides elicit anti‐citrullinated peptide antibodies (ACPAs) that are specific biomarkers of active disease in RA [22]. The molecular logic of this adaptive immune response is unclear. While the involvement of T‐helper cells is plausible, it is not known whether citrullination directly alters peptide affinity for disease‐associated HLAs (as is the case in CeD) or whether it influences the selection of proteolytic sites for epitope generation in the lysosome (The Val‐Cit peptide bond is a preferred target of lysosomal cathepsin B). As discussed below, the process of citrullination could also facilitate T cell activation by providing an efficient way for antigenic peptides to gain access to a complementary HLA, a journey that requires an extracellular peptide to cross two membrane barriers.

Like TG2, PADIs such as PADI2 and PADI4 are intracellular enzymes that can be released into the extracellular environment under certain conditions. For example, PADI4, which has a nuclear localization signal, is released by neutrophils into the extracellular space during a process known as NETosis [23]. Synovial fluids of RA patients contain elevated PADI2 and PADI4 levels accompanied by ACPAs. While the mechanism of ACPA development is unknown, their presence is strongly correlated with active disease, suggesting that citrullination plays a role in the breakdown of immune tolerance in RA [24].

### 2.3 Other Neo‐Epitope Forming PTMs

**SCHEME 3:**
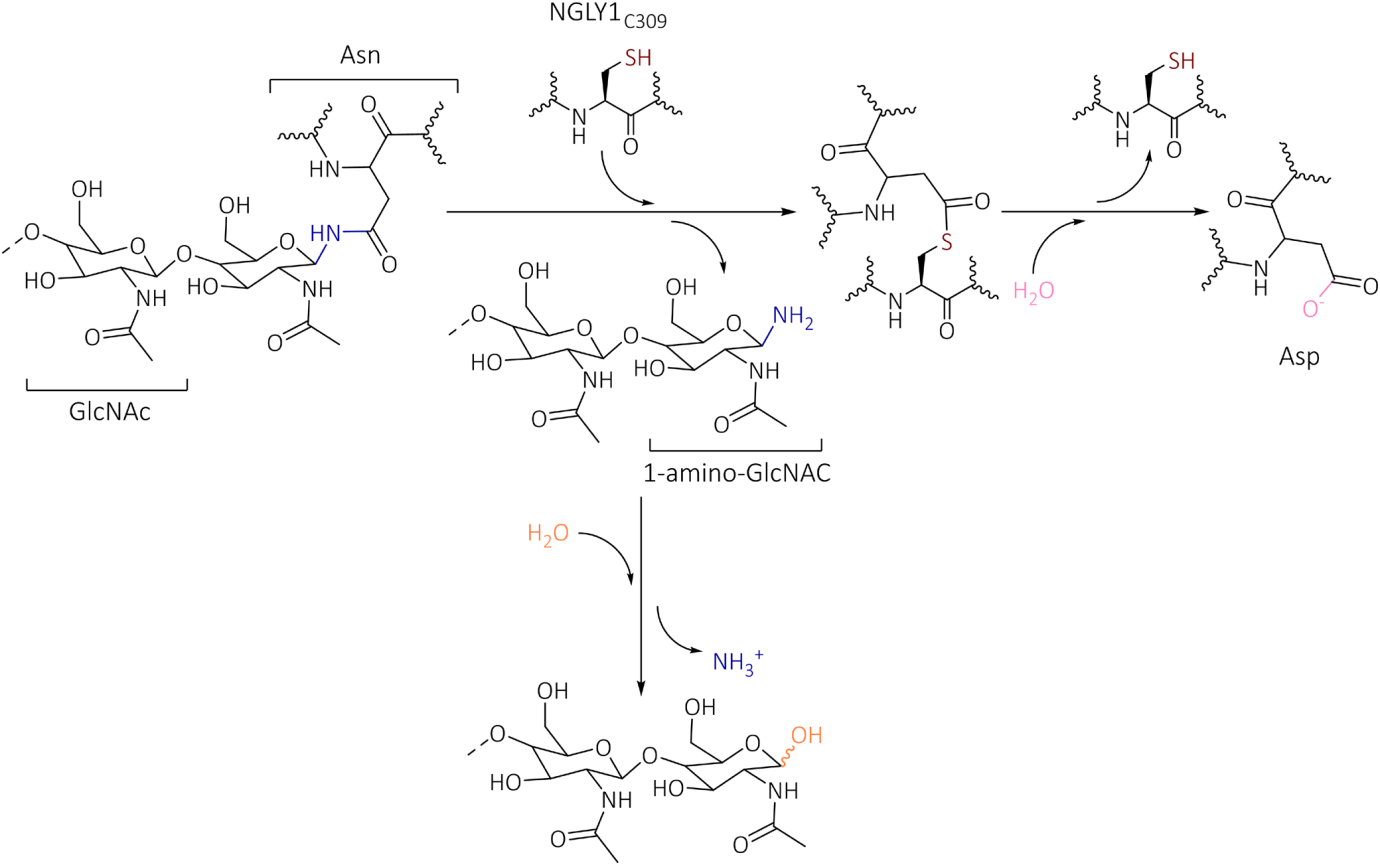
N‐glycan removal from glycoproteins by NGLY1.

Whereas both PTM biochemistry and T cell biology are mature fields of research unto themselves, the ability of PTMs to promote the appearance of neo‐epitope recognizing T cells is a relatively new concept. Both class I and II MHC‐driven T cell recognition can be influenced by PTMs. For an extensive listing of PTMs of potential interest in this regard, the reader is directed to an excellent review on the subject [25]. A particularly intriguing approach to the discovery of new paradigms in PTM‐driven autoimmunity is the clinical case report literature. For example, NGLY1 is an N‐glycanase that hydrolyzes Asn‐linked glycosides from glycoproteins (Scheme 3). While not Ca^2+^ dependent, it is evolutionarily and mechanistically related to TGs. Although most research into the physiologic relevance of NGLY1 has focused on a homozygous genetic deficiency of this enzyme, heterozygous variants have been associated with levodopa refractory parkinsonism with concomitant autoimmunity. In at least one such patient, treatment with tofacitinib (a well‐known T cell suppressive agent) resulted in improvement of motor symptoms as well as remission of autoimmune manifestations [26]. The potential link between an NGLY1‐catalyzed PTM and autoimmunity warrants further investigation in Parkinson's patients with a similar etiology.

## 3 Mechanisms for Antigen Transport Into the Lysosomes of Antigen Presenting Cells

Antigen‐presenting cells (APCs) play a central role in the initiation and propagation of adaptive immune responses. These specialized immune cells—including dendritic cells, macrophages, and B lymphocytes—are responsible for capturing antigens from the extracellular environment, transporting them to lysosomes, breaking them down into peptides, and presenting the resulting peptides to T lymphocytes in the context of specific MHC molecules. Through these processes, not only do APCs provide the essential link between extracellular antigens and the T cells that they activate, but they also strongly influence the identity of the antigens that are presented to T cells.

The generation of neo‐epitopes is particularly relevant for autoimmune pathogenesis. During thymic selection, developing T cells are exposed to a limited repertoire of self‐peptides presented by thymic APCs. If a putative host‐encoded peptide sequence (e.g., a post‐translationally modified neo‐peptide) is absent from this process of thymic selection, T cells capable of recognizing it may escape negative selection and thus persist in the naive immune repertoire. Under conditions favoring the appearance of these neo‐peptides, they can give rise to autoreactive T cells [27].

Beyond generating novel antigenic sequences, PTMs catalyzed within and around APCs can also influence the pathways by which native peptides are presented to T cells by these cells. Peptide uptake, lysosomal delivery, and proteolytic pruning are tightly regulated processes that determine which antigenic sequences are ultimately presented to T cells [28]. PTMs can affect antigen uptake by altering protein structure, solubility, aggregation state, proteolysis, and receptor‐binding properties. These changes may enhance recognition by specific cell‐surface receptors or modify the susceptibility of proteins to proteolytic degradation.

**FIGURE 2:**
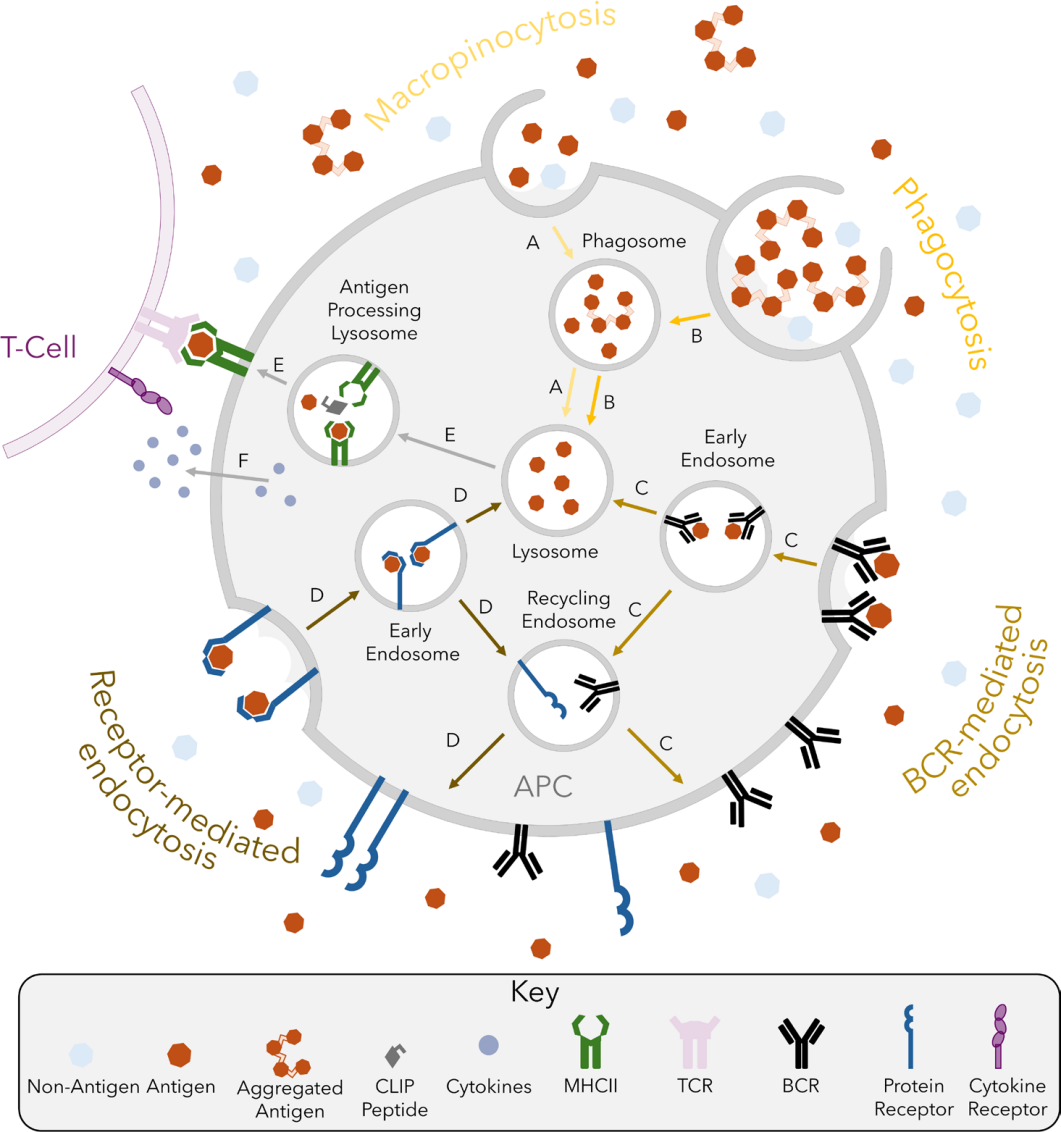
Endocytic mechanisms for antigen transport into APCs. Delivery of an antigen into the lysosome of an antigen presenting cell (APC) can occur via one of four types of pathways, each denoted by its own arrow letters and colors. (A) In macropinocytosis, sampling of the extracellular fluids by membrane reconstruction delivers the antigen to a phagosome, which is further processed into the lysosome. (B) Sampling of viruses, bacteria, and larger antigenic particles occurs via phagocytosis. Here too, the cargo is first processed in a phagosome followed by lysosome trafficking. (C) Antigen delivery by cell‐specific mechanisms such as recognition of a ligand by a B cell receptor (BCR) initiates endocytosis, delivering both BCR and antigen into early endosomes. The BCR is recycled to the cell surface, while the antigen is trafficked into the lysosome. (D) Receptor‐mediated endocytosis is a more general form of BCR‐mediated endocytosis, which allows for delivery based on antigen interactions with other receptors. When a ligand binds a receptor undergoing constitutive or ligand‐dependent endocytosis, both receptor and ligand are delivered into early endosomes. The receptor recycles to the cell surface, while the antigen is processed into the lysosome. (E) In the lysosome, the antigen replaces the CLIP peptide in the ligand‐binding groove of the MHCII, enabling presentation of the antigen‐MHC complex to T cells. (F) Upon activation of the T cell receptor, the APC releases cytokines that bind to the corresponding cytokine receptor on the T cell.

APCs employ three main mechanisms to capture extracellular antigens—phagocytosis, macropinocytosis, and receptor‐mediated endocytosis (Figure 2). Each pathway contributes distinctly to antigen acquisition and presentation. Phagocytosis enables the uptake of large particles such as microorganisms and apoptotic cells; macropinocytosis allows continuous sampling of extracellular fluid; and receptor‐mediated endocytosis provides a highly selective mechanism for internalizing specific antigens [29, 30]. These mechanisms are discussed below.

### 3.1 Phagocytosis

Phagocytosis allows cells to internalize large particles from their environments, including microorganisms, apoptotic cells, and cellular debris. This process is primarily performed by professional phagocytes such as macrophages and dendritic cells, which express a class of receptors that recognize pathogen‐associated molecular patterns (PAMPs) and damage‐associated molecular patterns (DAMPs).

The process of phagocytosis begins with the recognition of a target particle by a cell‐surface receptor. Engagement of the receptor triggers cytoskeletal rearrangements that lead to the extension of membrane protrusions around the particle. These protrusions eventually fuse to form a membrane‐bound vesicle known as a phagosome. The phagosome subsequently undergoes maturation through sequential fusion with endosomes and lysosomes, ultimately forming a phagolysosome [31].

Within the phagolysosome, the internalized material is exposed to an acidic environment and a diverse set of hydrolytic enzymes, which degrade proteins into peptide fragments that can be loaded onto MHC molecules. While phagocytosis provides an important mechanism to capture complex extracellular material for presentation to T cells, it does not appear to have evolved for APCs to extract antigens from individual molecules. Therefore, PTM‐derived antigens are taken up by phagocytosis in the context of larger particles that have undergone chemical modifications.

### 3.2 Macropinocytosis

Macropinocytosis facilitates non‐selective uptake of soluble molecules from the extracellular fluid. This process is particularly active in immature dendritic cells, which continuously sample their environment in search of potential antigens. It is initiated by actin‐driven membrane ruffling and results in the formation of large vesicles known as macropinosomes. Macropinosomes internalize substantial volumes of extracellular fluid. In intestinal mucosa, it enables APCs to monitor dietary antigens [32, 33]. For PTM‐derived antigens to be effectively sampled by this mechanism, the modification would likely have to change protein solubility or alter its interactions with extracellular matrix components.

### 3.3 Receptor‐Mediated Endocytosis

Receptor‐mediated endocytosis is the most selective and efficient uptake mechanism for antigen presentation by APCs because of its ability to harness molecular recognition of the antigen itself to enable it to cross two membrane barriers in the absence of more direct membrane transport mechanisms. It occurs when a cell‐surface receptor binds to a target antigen (either directly or via the intermediacy of a co‐receptor) and initiates internalization via clathrin‐coated pits. These vesicles then transport the bound antigens in a stepwise manner to lysosomes, where they are processed for presentation on MHC molecules. Meanwhile, the receptor is recycled back to the cell surface.

Many endocytic receptors, including scavenger receptors, complement receptors, Fc receptors, and C‐type lectin receptors, are expressed on professional APCs [30, 34]. Not only can their ligand recognition characteristics be influenced by PTMs, but in some cases, the processes of PTM generation and endocytosis are coupled. For example, in CeD, gluten antigens bind to catalytically active TG2 in the extracellular environment [35]. The product of TG2‐catalyzed deamidation must be transported to the lysosome where HLA‐DQ2/DQ8 are present. Receptor‐mediated endocytosis is clearly the preferred mechanism for this outcome. At least two distinct (non‐exclusive) mechanisms have been proposed for coupling the PTM with endocytosis; each mechanism exploits a unique biological characteristic of the enabling APC.

In one model, differentiated B cells with receptors (BCRs) that exhibit high affinity for deamidated gluten peptides (DGPs) facilitate access of these neo‐antigens to HLA‐DQ2/DQ8 (Figure 2); in turn, Th1 cells that recognize the HLA‐DGP complex promote differentiation of this B cell into a plasma cell that secretes high‐affinity anti‐DGP antibodies [36]. A variation on this theme postulates that, when TG2 forms a covalent complex with an antigenic gluten peptide, this transient species is recognized by a germline BCR with specificity for the TG2 protein [37]. If endocytosis occurs on a timescale faster than the second half‐reaction of TG2 (Scheme 1), then the resulting DGPs can be delivered to the lysosome of this B cell, leading to a plasma cell that secretes an anti‐TG2 autoantibody. While anti‐TG2 autoantibodies are in fact diagnostic hallmarks of CeD, the ability of antibodies to spatially segregate the two half‐reactions of TG2 in distinct subcellular compartments has not been demonstrated.

An alternate model for coupling TG2‐catalyzed PTM to endocytosis of gluten antigens is based on the observation that antigenic gluten peptides undergo efficient TG2‐dependent endocytosis in cells expressing low‐density lipoprotein receptor‐related protein 1 (LRP1), a highly expressed receptor on APCs of myeloid but not lymphoid lineages [1, 2, 38]. In this model, TG2 is a co‐receptor for LRP1. This TG2/LRP1 pathway for receptor‐mediated endocytosis of gluten peptides appears to be especially active in dendritic cell subpopulations and is the focus of the following section.

## 4 TG2/LRP1 Pathway for Coupled Deamidation and HLA Loading of Gluten Antigens

TG2 catalyzed post‐translational deamidation of gluten peptides is widely recognized as a causative event in the onset and lifelong persistence of CeD autoimmunity. Deamidation introduces a negative charge at a specific position in the DGP product, which enhances its affinity to HLA‐DQ2, the primary CeD‐associated MHC protein [6, 9, 39, 40] (An analogous PTM enhances the affinity of other gluten peptides to HLA‐DQ8, which is associated with a small minority of CeD cases).

Notwithstanding biochemical clarity, two key questions have eluded satisfactory answers in the context of the role of this PTM in CeD pathogenesis. First, what is the source of TG2 activity in the celiac small intestine? And second, assuming this enzymatic activity is extracellular, how is its product efficiently delivered into lysosomal HLA‐DQ2/DQ8? The former question is relevant because, although TG2 is an abundant protein in the ECM of the healthy mammalian small intestine, it is catalytically inactive [2, 41]. The latter question is important because the Michaelis constants (*K*_*M*_) of TG2 for all known antigenic gluten peptides are more than an order of magnitude higher than their anticipated intestinal concentrations due to gluten consumption in amounts that are toxic to a CeD patient (Oral gluten induces an inflammatory T cell response in CeD patients at an ED_50_ dose of 111 mg, ~5% of the gluten content in a slice of bread [42]). Under such conditions, solution‐phase DGP formation is unlikely to be an efficient source of neo‐antigens. The TG2/LRP1 pathway provides a compelling answer to both these questions.

**FIGURE 3:**
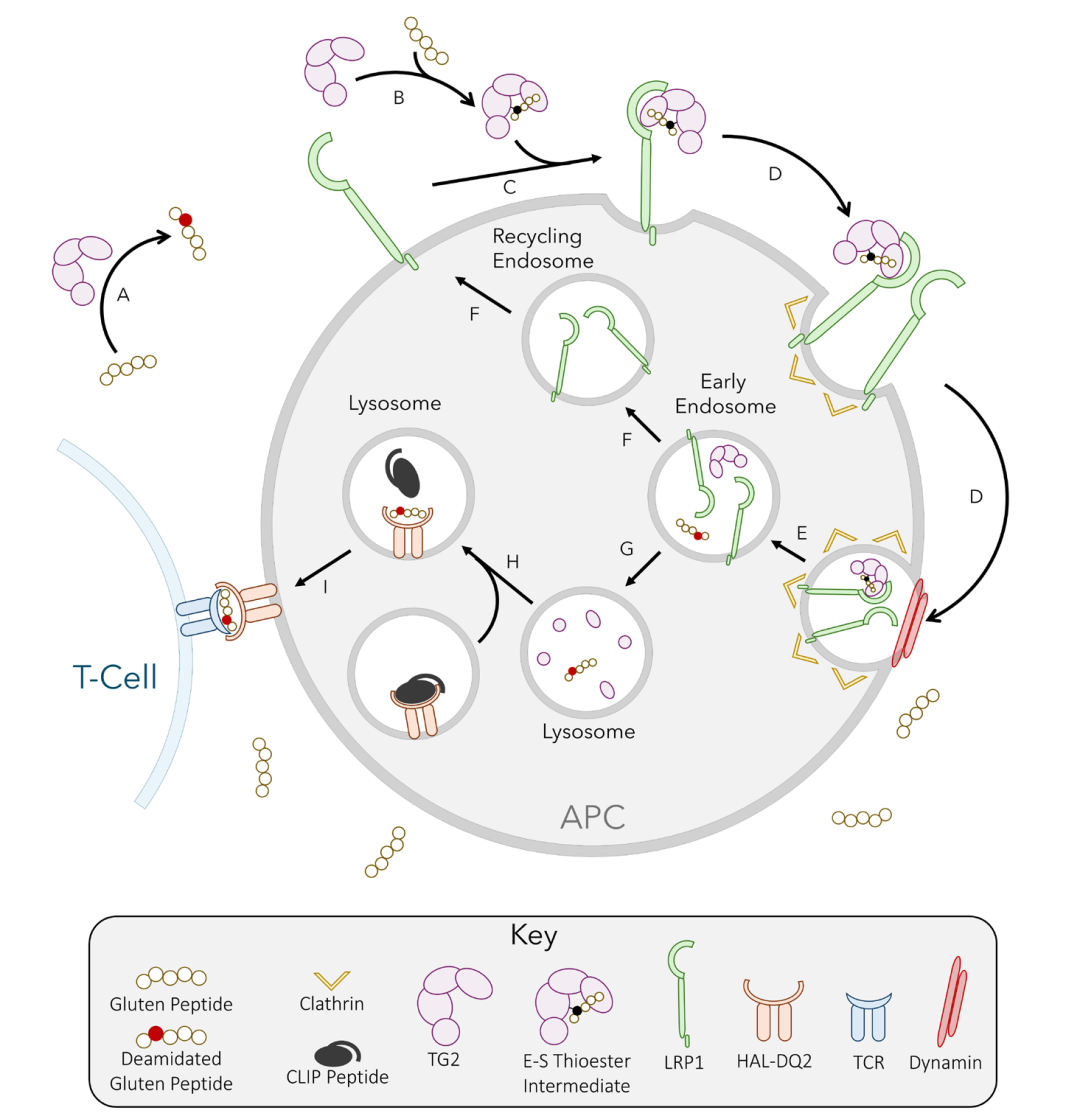
TG2/LRP1 pathway for gluten antigen presentation by dendritic cells. The overall pathway is depicted in a stepwise manner with a letter assigned to each step. (A) Extracellular TG2 can catalyze PTMs on its peptide substrates by converting selected Gln residues into Glu (red circle). (B) Because peptide deamidation requires TG2 to first form a covalent intermediate with its substrate (black circle), (C) for certain substrates such as gluten‐derived antigens this covalent intermediate is bound by the LRP1 endocytic receptor. LRP1 binding stabilizes the TG2‐peptide adduct by preventing the hydrolytic reaction from occurring. (D) Engagement of multiple (occupied or unoccupied) LRP1 receptors initiates clathrin dependent endocytosis, a process that entails membrane indentation and budding with assistance from motor proteins such as dynamin, (E) delivering LRP1 and the TG2‐gluten covalent intermediate into early endosomes. Endosome acidification allows TG2 to be released from LRP1 and the second half (hydrolysis) of the catalytic cycle to be completed, resulting in the release of a deamidated gluten peptide in the endosomal environment. (F) LRP1 is recycled back to the cell surface via a recycling endosome while (G) TG2 and the deamidated gluten peptide are trafficked into the lysosome. There, TG2 is degraded by proteases while (H) the deamidated antigenic peptide encounters HLA‐DQ2 (or HLA‐DQ8). For the peptide to bind to this MHCII protein, it must displace the endogenous CLIP peptide. (I) The resulting complex of HLA‐DQ2 bound to the deamidated gluten peptide is transported to the surface of the APC for T cell presentation.

As shown in Figure 3, certain cells of the myeloid lineage, most notably dendritic cells, harbor catalytically active TG2 near their cell surfaces [43]. While membrane‐associated TG2 is a common feature of mammalian cells, it typically lacks detectable enzyme activity. In contrast, dendritic cells exhibit clusters of surface TG2 activity. Although the physical nature of this catalytically active TG2 is unknown, one must presume that its two Ca^2+^ binding sites are occupied and its inactivating C370‐C371 disulfide bond is in a reduced state [41, 44]. When TG2 on the surface of dendritic cells binds to a gluten‐derived peptide substrate, it is recognized by LRP1, a single‐pass transmembrane endocytic receptor known to transport diverse extracellular ligands from the ECM into the lysosome. Recent work in our laboratory has established that this interaction is centered on one of the two C‐terminal b‐barrel domains of TG2.

Two features of the interaction between substrate‐bound TG2 and LRP1 are noteworthy. First, not all TG2 substrates (or active site inhibitors) can elicit this protein–protein interaction. For example, whereas the pentapeptide sequence PQLPF found in many antigenic gluten peptides is highly effective at eliciting this interaction, a widely used reference TG2 substrate ZQG (Z = benzyloxycarbonyl) is not [12]. Second, upon docking to LRP1, the thioester product of the first half‐reaction (Scheme 1) appears to be catalytically “frozen” for the duration of receptor‐mediated endocytosis (~10 min) without losing its ability to undergo the second half‐reaction; ordinarily the latter half‐reaction occurs in ~1 s. An atomic‐level mechanistic explanation for neither of these remarkable properties of TG2 is lacking.

Upon undergoing receptor‐mediated endocytosis, the ternary complex between LRP1, TG2, and a peptide substrate rapidly decouples, presumably in response to the acidic environment of endosomes. The LRP1 receptor is recycled to the cell surface, while TG2 and the deamidated product are shuttled to the lysosome [45]. Overall, the TG2/LRP1 pathway couples antigen capture with PTM, facilitating the efficient delivery of neo‐epitopes to HLA‐DQ2/DQ8.

Several lines of evidence support the relevance of the TG2/LRP1 pathway in CeD pathogenesis. First and foremost, intestinal biopsies obtained from gluten challenged CeD patients show increased numbers of CD14^+^CD11c^+^ dendritic cells in lamina propria [46]. These dendritic cells are known to exhibit strong antigen‐presenting capacity and presumably play a key role in activating gluten‐specific CD4^+^ T cells. Second, in a healthy mouse intestine, CD103^+^ dendritic cells are the predominant cell type that exhibits TG2/LRP1 pathway activity; an extensive body of literature supports their role in sampling dietary antigens and presenting them to T cells [2]. Third, in HLA‐DQ2 transgenic mice, CD103^+^ dendritic cells loaded with orally administered DGPs migrate from the intestinal lamina propria and Peyer's patches into mesenteric lymph nodes where they engage T cells; in turn, gluten antigen presentation to T cells upregulates TG2 activity in the intestinal mucosa [2]. This mutual gluten‐dependent reinforcement between intestinal HLA‐DQ2 activity and TG2 activity has been widely anticipated but never been directly demonstrated. Last but not least, the strong correlation between gluten peptide antigenicity in CeD and their ability to activate LRP1‐dependent endocytosis is suggestive of the pathogenic relevance of the TG2/LRP1 pathway.

A fluorescent small molecule reagent, HB‐230 (BioTechne catalog # 8831) has proven to be a particularly effective tool for labeling cells with an active TG2/LRP1 pathway. Using this tool, the TG2/LRP1 pathway has been found to exhibit remarkable cell selectivity in mammals [2]. Among white blood cells derived from rodents and humans, the highest activity is observed in neutrophils and monocytes with virtually no activity in T‐ or B‐lymphocytes. When bone marrow‐derived monocytes are differentiated into dendritic cells or macrophages in vitro, the highest TG2/LRP1 pathway activity is observed in immature cells. In solid organs too, very few cells exhibit detectable pathway activity. For example, epithelial and endothelial cells in the intestine, lung or kidney of healthy mammals are not labeled by HB‐230, although tissue‐resident dendritic cells and some stromal fibroblasts are labeled. In response to inflammation or injury, not only is the TG2/LRP1 pathway rapidly activated in an expanding number of cells in the affected organ, but HB‐230 is also observed to label proteins in the ECM^2^. Because TG2 is known to be associated with collagen and fibronectin, presumably this reflects an increased rate of TG2 release from dendritic cells and fibroblasts [2, 47].

## 5 Implications of the TG2/LRP1 Pathway for Other Diseases

**FIGURE 4:**
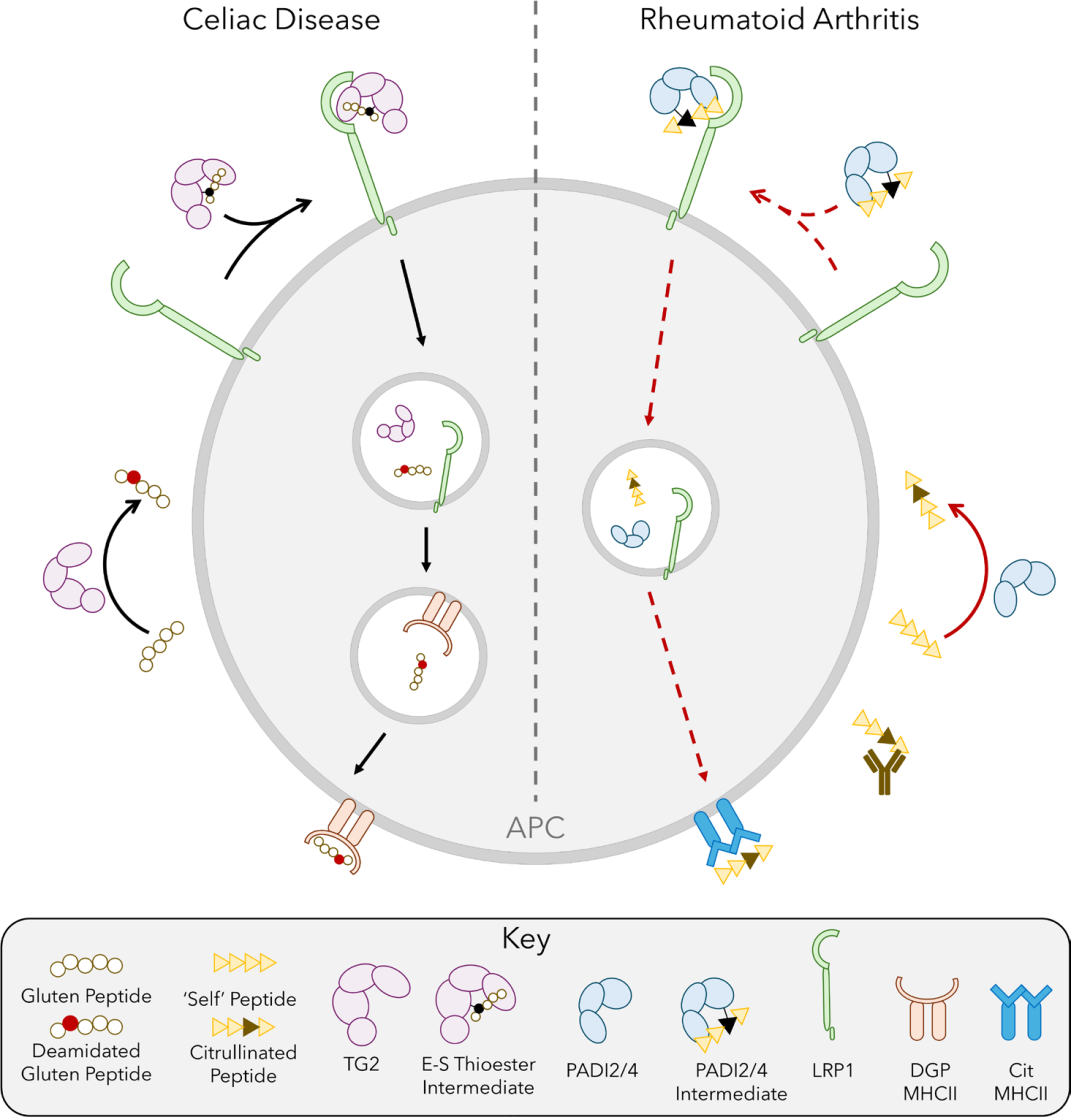
Parallels between celiac disease (CeD) and rheumatoid arthritis (RA). Delivery of an antigenic peptide via the TG2/LRP1 pathway is shown by black arrows to the left. TG2 catalyzes the deamidation of gluten peptides; antibodies against deamidated gluten peptides (DGPs) are well‐known biomarkers of CeD. The intermediate state in the TG2‐catalyzed deamidation reaction allows the enzyme to engage with the LRP1 endocytic receptor (Figure 3). Upon lysosomal delivery, the DGP is available for MHCII‐dependent antigen presentation. An entirely analogous mechanism for neo‐antigen presentation in RA is shown in red. PADI catalyzes the formation of citrullinated peptides via a Cys‐bound covalent intermediate. Antibodies that recognize citrullinated peptides are well‐known markers of RA. A proposed mechanism for citrullinated antigen delivery to MHCII in RA is shown by the dashed red arrows, where the PADI‐bound covalent intermediate engages LRP1 to deliver a citrullinated peptide in the early endosome. A key prediction of this model is that PADI is capable of engaging the LRP1 receptor through specific protein–protein interactions.

The discovery of the TG2/LRP1 pathway as well as recognition of its high degree of cell selectivity offers a new window to our understanding of antigen presentation in CeD. Beyond CeD, its relevance to other immune disorders is worth considering. In particular, could the LRP1 receptor coopt other PTM‐catalyzing extracellular enzymes beyond TG2 as co‐receptors? For example, like CeD, RA is associated with circulating antibodies against post‐translationally modified antigens (Figure 4). Additionally, these antigens, derived from enzymatic PTMs, have greater affinity to the HLA molecule genetically associated with HLA‐DRB1*0401, an RA‐associated HLA [48]. If PADI2 and/or PADI4 can engage LRP1 in a substrate‐dependent manner, then one can envision how citrullination of self‐peptides would transform them into potent neo‐antigens.

Beyond autoimmune diseases, the inherent restriction of the TG2/LRP1 pathway activity to fibroblasts and cells of the myeloid lineage also motivates investigation into its role in organ fibrosis, where TGFb is thought to be a master‐signal. When TGFb is released by injured cells and infiltrating monocytes that sense the injury, it induces proliferation of fibroblasts and causes them to produce excessive fibrotic tissue. If activity of the TG2/LRP1 pathway is upregulated in response to pro‐fibrotic injury, then it could counterbalance fibrosis by lysosomal delivery and degradation of excess collagen and fibronectin. In such a scenario, runaway fibrosis could be envisioned as the inability of proteostatic processes like the TG2/LRP1 pathway to keep up.

## Funding

This work was supported by National Institutes of Health (R01 DK063158).

## Conflicts of Interest

The authors declare no conflicts of interest.

## Data Availability

Data sharing not applicable to this article as no datasets were generated or analyzed during the current study.

## References

[1] E. Loppinet , H. A. Besser , A. S. Sewa , F. C. Yang , B. Jabri , and C. Khosla , “LRP‐1 Links Post‐Translational Modifications to Efficient Presentation of Celiac Disease‐Specific T Cell Antigens,” Cell Chemical Biology 30, no. 1 (2023): 55–68.e10, 10.1016/j.chembiol.2022.12.002. 36608691 PMC9868102

[2] F. C. Yang , H. A. Besser , H. R. Chun , et al., “A Dendritic Cell Population Responsible for Transglutaminase 2–Mediated Gluten Antigen Presentation in Celiac Disease,” JCI Insight 10, no. 19 (2025): e196102, 10.1172/jci.insight.196102. 40875488 PMC12513477

[3] L. Lorand and R. M. Graham , “Transglutaminases: Crosslinking Enzymes With Pleiotropic Functions,” Nature Reviews. Molecular Cell Biology 4, no. 2 (2003): 140–156, 10.1038/nrm1014. 12563291

[4] S. E. Iismaa , B. M. Mearns , L. Lorand , and R. M. Graham , “Transglutaminases and Disease: Lessons From Genetically Engineered Mouse Models and Inherited Disorders,” Physiological Reviews 89, no. 3 (2009): 991–1023, 10.1152/physrev.00044.2008. 19584319

[5] Z. Keresztessy , É. Csősz , J. Hársfalvi , et al., “Phage Display Selection of Efficient Glutamine‐Donor Substrate Peptides for Transglutaminase 2,” Protein Science 15, no. 11 (2006): 2466–2480, 10.1110/ps.051818406. 17075129 PMC2242420

[6] S. W. Qiao , E. Bergseng , Ø. Molberg , et al., “Antigen Presentation to Celiac Lesion‐Derived T Cells of a 33‐Mer Gliadin Peptide Naturally Formed by Gastrointestinal Digestion,” Journal of Immunology 173, no. 3 (2004): 1757–1762, 10.4049/jimmunol.173.3.1757. 15265905

[7] S. Martucciello , S. Sposito , C. Esposito , G. Paolella , and I. Caputo , “Interplay Between Type 2 Transglutaminase (TG2), Gliadin Peptide 31‐43 and Anti‐TG2 Antibodies in Celiac Disease,” International Journal of Molecular Sciences 21, no. 10 (2020): 3673, 10.3390/ijms21103673. 32456177 PMC7279455

[8] B. A. Palanski , N. Weng , L. Zhang , et al., “An Efficient Urine Peptidomics Workflow Identifies Chemically Defined Dietary Gluten Peptides From Patients With Celiac Disease,” Nature Communications 13, no. 1 (2022): 888, 10.1038/s41467-022-28353-1. PMC8850430 35173144

[9] H. Arentz‐Hansen , S. N. Mcadam , Ø. Molberg , et al., “Celiac Lesion T Cells Recognize Epitopes That Cluster in Regions of Gliadins Rich in Proline Residues,” Gastroenterology 123, no. 3 (2002): 803–809, 10.1053/gast.2002.35381. 12198706

[10] S. Tollefsen , “HLA‐DQ2 and ‐DQ8 Signatures of Gluten T Cell Epitopes in Celiac Disease,” Journal of Clinical Investigation 116, no. 8 (2006): 2226–2236, 10.1172/JCI27620. 16878175 PMC1518792

[11] J. Xia , M. Siegel , E. Bergseng , L. M. Sollid , and C. Khosla , “Inhibition of HLA‐DQ2‐Mediated Antigen Presentation by Analogues of a High Affinity 33‐Residue Peptide From α2‐Gliadin,” Journal of the American Chemical Society 128, no. 6 (2006): 1859–1867, 10.1021/ja056423o. 16464085 PMC2597451

[12] A. S. Sewa , H. A. Besser , I. I. Mathews , and C. Khosla , “Structural and Mechanistic Analysis of Ca^2+^ −Dependent Regulation of Transglutaminase 2 Activity Using a Ca^2+^ −Bound Intermediate State,” Proceedings of the National Academy of Sciences of the United States of America 121, no. 28 (2024): e2407066121, 10.1073/pnas.2407066121. 38959038 PMC11252922

[13] R. Király , É. Csősz , T. Kurtán , et al., “Functional Significance of Five Noncanonical Ca^2+^ −Binding Sites of Human Transglutaminase 2 Characterized by Site‐Directed Mutagenesis,” FEBS Journal 276, no. 23 (2009): 7083–7096, 10.1111/j.1742-4658.2009.07420.x. 19878304

[14] R. Tilvawala and P. R. Thompson , “Peptidyl Arginine Deiminases: Detection and Functional Analysis of Protein Citrullination,” Current Opinion in Structural Biology 59 (2019): 205–215, 10.1016/j.sbi.2019.01.024. 30833201 PMC6717693

[15] K. Arita , H. Hashimoto , T. Shimizu , K. Nakashima , M. Yamada , and M. Sato , “Structural Basis for Ca2+−Induced Activation of Human PAD4,” Nature Structural & Molecular Biology 11, no. 8 (2004): 777–783, 10.1038/nsmb799. 15247907

[16] S. Saijo , A. Nagai , S. Kinjo , et al., “Monomeric Form of Peptidylarginine Deiminase Type I Revealed by X‐Ray Crystallography and Small‐Angle X‐Ray Scattering,” Journal of Molecular Biology 428, no. 15 (2016): 3058–3073, 10.1016/j.jmb.2016.06.018. 27393304

[17] D. J. Slade , P. Fang , C. J. Dreyton , et al., “Protein Arginine Deiminase 2 Binds Calcium in an Ordered Fashion: Implications for Inhibitor Design,” ACS Chemical Biology 10, no. 4 (2015): 1043–1053, 10.1021/cb500933j. 25621824 PMC4569063

[18] C. Zhu , C. Liu , and Z. Chai , “Role of the PADI Family in Inflammatory Autoimmune Diseases and Cancers: A Systematic Review,” Frontiers in Immunology 14 (2023): 1115794, 10.3389/fimmu.2023.1115794. 37020554 PMC10067674

[19] C. Tanikawa , K. Ueda , A. Suzuki , et al., “Citrullination of RGG Motifs in FET Proteins by PAD4 Regulates Protein Aggregation and ALS Susceptibility,” Cell Reports 22, no. 6 (2018): 1473–1483, 10.1016/j.celrep.2018.01.031. 29425503

[20] A. S. Rebak , I. A. Hendriks , J. D. Elsborg , et al., “A Quantitative and Site‐Specific Atlas of the Citrullinome Reveals Widespread Existence of Citrullination and Insights Into PADI4 Substrates,” Nature Structural & Molecular Biology 31, no. 6 (2024): 977–995, 10.1038/s41594-024-01214-9. PMC11189309 38321148

[21] C. J. Dreyton , B. Knuckley , J. E. Jones , D. M. Lewallen , and P. R. Thompson , “Mechanistic Studies of Protein Arginine Deiminase 2: Evidence for a Substrate‐Assisted Mechanism,” Biochemistry 53, no. 27 (2014): 4426–4433, 10.1021/bi500554b. 24989433 PMC4100781

[22] D. Damgaard , M. Bawadekar , L. Senolt , A. Stensballe , M. A. Shelef , and C. H. Nielsen , “Relative Efficiencies of Peptidylarginine Deiminase 2 and 4 in Generating Target Sites for Anti‐Citrullinated Protein Antibodies in Fibrinogen, Alpha‐Enolase and Histone H3,” PLoS One 13, no. 8 (2018): e0203214, 10.1371/journal.pone.0203214. 30161253 PMC6117052

[23] H. Yao , G. Cao , Z. Liu , et al., “Inhibition of Netosis With PAD Inhibitor Attenuates Endotoxin Shock Induced Systemic Inflammation,” International Journal of Molecular Sciences 23, no. 21 (2022): 13264, 10.3390/ijms232113264. 36362052 PMC9655899

[24] W. Kurowska , E. H. Kuca‐Warnawin , A. Radzikowska , and W. Maśliński , “The Role of Anti‐Citrullinated Protein Antibodies (ACPA) in the Pathogenesis of Rheumatoid Arthritis,” Central European Journal of Immunology 42, no. 4 (2017): 390–398, 10.5114/ceji.2017.72807. 29472818 PMC5820977

[25] A. S. De Wit , F. Bianchi , and G. Van Den Bogaart , “Antigen Presentation of Post‐Translationally Modified Peptides in Major Histocompatibility Complexes,” Immunology and Cell Biology 103, no. 2 (2025): 161–177, 10.1111/imcb.12839. 39609891 PMC11792782

[26] N. Reina‐Llompart , S. Serrano‐López , G. Torres‐Iglesias , and J. Álvarez‐Troncoso , “Tofacitinib Improves Motor Symptoms in Parkinsonism Associated With a Heterozygous NGLY1 Variant and Autoimmune Disease,” European Journal of Case Reports in Internal Medicine 12, no. 11 (2025), 10.12890/2025_005886. PMC12604884 41229635

[27] M. Rojas , Y. Acosta‐Ampudia , L. S. Heuer , et al., “Antigen‐Specific T Cells and Autoimmunity,” Journal of Autoimmunity 148 (2024): 103303, 10.1016/j.jaut.2024.103303. 39141985

[28] L. Delamarre , M. Pack , H. Chang , I. Mellman , and E. S. Trombetta , “Differential Lysosomal Proteolysis in Antigen‐Presenting Cells Determines Antigen Fate,” Science 307, no. 5715 (2005): 1630–1634, 10.1126/science.1108003. 15761154

[29] Z. Liu and P. A. Roche , “Macropinocytosis in Phagocytes: Regulation of MHC Class‐II‐Restricted Antigen Presentation in Dendritic Cells,” Frontiers in Physiology 6 (2015), 10.3389/fphys.2015.00001. PMC4311620 25688210

[30] C. D. Platt , J. K. Ma , C. Chalouni , et al., “Mature Dendritic Cells Use Endocytic Receptors to Capture and Present Antigens,” Proceedings of the National Academy of Sciences 107, no. 9 (2010): 4287–4292, 10.1073/pnas.0910609107. PMC2840134 20142498

[31] A. R. Mantegazza , J. G. Magalhaes , S. Amigorena , and M. S. Marks , “Presentation of Phagocytosed Antigens by MHC Class I and II,” Traffic 14, no. 2 (2013): 135–152, 10.1111/tra.12026. 23127154 PMC3538944

[32] V. Cabric , Y. F. Parisotto , T. Park , et al., “A Wave of Thetis Cells Imparts Tolerance to Food Antigens Early in Life,” Science 389, no. 6757 (2025): 268–274. 40373113 10.1126/science.adp0535 PMC13245555

[33] J. Abramson , J. Dobeš , M. Lyu , and G. F. Sonnenberg , “The Emerging Family of RORγt+ Antigen‐Presenting Cells,” Nature Reviews. Immunology 24, no. 1 (2024): 64–77, 10.1038/s41577-023-00906-5. PMC10844842 37479834

[34] W. S. Garrett , L. M. Chen , R. Kroschewski , et al., “Developmental Control of Endocytosis in Dendritic Cells by Cdc42,” Cell 102, no. 3 (2000): 325–334, 10.1016/S0092-8674(00)00038-6. 10975523

[35] M. Siegel , P. Strnad , R. E. Watts , et al., “Extracellular Transglutaminase 2 Is Catalytically Inactive, but Is Transiently Activated Upon Tissue Injury,” PLoS One 3, no. 3 (2008): e1861, 10.1371/journal.pone.0001861. 18365016 PMC2267210

[36] I. Lindeman , C. Zhou , L. M. Eggesbø , et al., “Longevity, Clonal Relationship, and Transcriptional Program of Celiac Disease–Specific Plasma Cells,” Journal of Experimental Medicine 218, no. 2 (2021): e20200852, 10.1084/jem.20200852. 33095260 PMC7590513

[37] C. B. Lindstad , A. E. Dewan , J. Stamnaes , L. M. Sollid , and M. F. Du Pré , “TG2‐Gluten Complexes as Antigens for Gluten‐Specific and Transglutaminase‐2 Specific B Cells in Celiac Disease,” PLoS One 16, no. 11 (2021): e0259082, 10.1371/journal.pone.0259082. 34731200 PMC8565743

[38] S. K. Moestrup , J. Gliemann , and G. Pallesen , “Distribution of the α2‐Macroglobulin Receptor/Low Density Lipoprotein Receptor‐Related Protein in Human Tissues,” Cell and Tissue Research 269, no. 3 (1992): 375–382, 10.1007/BF00353892. 1423505

[39] B. Fleckenstein , Ø. Molberg , S. W. Qiao , et al., “Gliadin T Cell Epitope Selection by Tissue Transglutaminase in Celiac Disease,” Journal of Biological Chemistry 277, no. 37 (2002): 34109–34116, 10.1074/jbc.M204521200. 12093810

[40] C. Y. Kim , H. Quarsten , E. Bergseng , C. Khosla , and L. M. Sollid , “Structural Basis for HLA‐DQ2‐Mediated Presentation of Gluten Epitopes in Celiac Disease,” Proceedings of the National Academy of Sciences 101, no. 12 (2004): 4175–4179, 10.1073/pnas.0306885101. PMC384714 15020763

[41] X. Jin , J. Stamnaes , C. Klöck , T. R. DiRaimondo , L. M. Sollid , and C. Khosla , “Activation of Extracellular Transglutaminase 2 by Thioredoxin,” Journal of Biological Chemistry 286, no. 43 (2011): 37866–37873, 10.1074/jbc.M111.287490. 21908620 PMC3199528

[42] A. J. M. Daveson , E. Craig , A. Vitak , et al., “A Randomized Double‐Blind, Placebo‐Controlled Dose‐Response Study to Assess the Gluten Threshold Dose in Celiac Disease,” Gastroenterology (2026): S0016508526002647, 10.1053/j.gastro.2026.03.011. 41903816

[43] M. Ráki , K. W. Schjetne , J. Stamnaes , et al., “Surface Expression of Transglutaminase 2 by Dendritic Cells and Its Potential Role for Uptake and Presentation of Gluten Peptides to T Cells,” Scandinavian Journal of Immunology 65, no. 3 (2007): 213–220, 10.1111/j.1365-3083.2006.01881.x. 17309775

[44] J. Stamnaes , D. M. Pinkas , B. Fleckenstein , C. Khosla , and L. M. Sollid , “Redox Regulation of Transglutaminase 2 Activity,” Journal of Biological Chemistry 285, no. 33 (2010): 25402–25409, 10.1074/jbc.M109.097162. 20547769 PMC2919103

[45] E. A. Zemskov , I. Mikhailenko , D. K. Strickland , and A. M. Belkin , “Cell‐Surface Transglutaminase Undergoes Internalization and Lysosomal Degradation: An Essential Role for LRP1,” Journal of Cell Science 120, no. 18 (2007): 3188–3199, 10.1242/jcs.010397. 17711877

[46] A. C. R. Beitnes , M. Ráki , M. Brottveit , K. E. A. Lundin , F. L. Jahnsen , and L. M. Sollid , “Rapid Accumulation of CD14+CD11c+ Dendritic Cells in Gut Mucosa of Celiac Disease After In Vivo Gluten Challenge,” PLoS One 7, no. 3 (2012): e33556, 10.1371/journal.pone.0033556. 22438948 PMC3306402

[47] I. Cardoso , J. Stamnaes , J. T. Andersen , G. Melino , R. Iversen , and L. M. Sollid , “Transglutaminase 2 Interactions With Extracellular Matrix Proteins as Probed With Celiac Disease Autoantibodies,” FEBS Journal 282, no. 11 (2015): 2063–2075, 10.1111/febs.13276. 25808416

[48] S. C. Law , S. Street , C. H. A. Yu , et al., “T‐Cell Autoreactivity to Citrullinated Autoantigenic Peptides in Rheumatoid Arthritis Patients Carrying HLA‐DRB1 Shared Epitope Alleles,” Arthritis Research & Therapy 14, no. 3 (2012): R118, 10.1186/ar3848. 22594821 PMC3446499

